# The relationship between specialized pro-resolving lipid mediators, morbid obesity and weight loss after bariatric surgery

**DOI:** 10.1038/s41598-020-75353-6

**Published:** 2020-11-18

**Authors:** Fabian Schulte, Abdul Aziz Asbeutah, Peter N. Benotti, G. Craig Wood, Christopher Still, Bruce R. Bistrian, Markus Hardt, Francine K. Welty

**Affiliations:** 1 Forsyth Institute, Cambridge, MA USA; 2 Department of Developmental Biology, Harvard School of Dental Medicine, Boston, MA USA; 3 Division of Cardiology, Beth Israel Deaconess Medical Center, Harvard Medical School, 330 Brookline Avenue, SL 423, Boston, MA 02215 USA; 4 Geisinger Obesity Institute, Danville, PA USA; 5 Department of Medicine, Beth Israel Deaconess Medical Center, Harvard Medical School, Boston, MA USA; 6 Present Address: Department of Medicine, University of Tennessee Health Science Center, Memphis, TN USA

**Keywords:** Biomarkers, Medical research

## Abstract

Obesity and diabetes are associated with chronic inflammation. Specialized pro-resolving lipid mediators (SPMs)—resolvins (Rv), protectins (PD) and maresins (MaR)—actively resolve inflammation. Bariatric surgery achieves remission of diabetes, but mechanisms are unclear. We measured SPMs and proinflammatory eicosanoid levels using liquid chromatography-tandem mass spectrometry in 29 morbidly obese subjects (13 with diabetes) and 15 nondiabetic, mildly obese subjects. Compared to the mildly obese, the morbidly obese had higher levels of SPMs—RvD3, RvD4 and PD1—and white blood cells (WBC) and platelets. Post-surgery, SPM and platelet levels decreased in morbidly obese nondiabetic subjects but not in diabetic subjects, suggesting continued inflammation. Despite similar weight reductions 1 year after surgery (44.6% vs. 46.6%), 8 diabetes remitters had significant reductions in WBC and platelet counts whereas five non-remitters did not. Remitters had a 58.2% decrease (*p* = 0.03) in 14-HDHA, a maresin pathway marker; non-remitters had an 875.7% increase in 14-HDHA but a 36.9% decrease in MaR1 to a median of 0. In conclusion, higher levels of RvD3, PD1 and their pathway marker, 17-HDHA, are markers of leukocyte activation and inflammation in morbid obesity and diabetes and diminish with weight loss in nondiabetic but not diabetic subjects, possibly representing sustained inflammation in the latter. Lack of diabetes remission after surgically-induced weight loss may be associated with reduced ability to produce MaR1 and sustained inflammation.

## Introduction

Visceral adiposity is the major risk factor responsible for the development of insulin resistance common to metabolic syndrome and type 2 diabetes^1^. White adipose tissue releases more than 50 different adipocytokines including the cytokines, tumor necrosis factor (TNF)-α and interleukin (IL)-6. TNF-α signaling activates intracellular kinases, c-Jun N-terminal kinase and IκB kinase, leading to increased serine phosphorylation of insulin receptor substrate-1 (IRS-1) which impairs insulin signaling^2,3^, causing insulin resistance that inhibits the transport of glucose into cells^4,5^.

Inflammation is a protective host response which protects against tissue invasion and injury and initiates healing to minimize tissue damage and restore normal function^6^. In the initiation phase, elevated free fatty acids activate the proinflammatory pathway by upregulating cyclooxygenase (COX) which converts arachidonic acid to proinflammatory, prothrombotic and vasoactive eicosanoids that include prostaglandin [PG] D_2_, PGE_2_, PGF_2α_, thromboxane [Tx] A_2_ and TxB_2_, and via 5-lipoxygenase (LOX) which converts arachidonic acid to the pro-inflammatory leukotriene (LT) B_4_, a potent chemoattractant that recruits neutrophils into tissue to remove necrotic debris and apoptotic cells^7,8^. Neutrophils release TNF-α and IL-6 which cause insulin resistance directly via serine phosphorylation of IRS-1. Second, neutrophils upregulate COX and LOX enzymes, which increase production of LTB_4_^9,10^, leading to recruitment of more neutrophils, thus prolonging exposure to elevated cytokine levels and furthering insulin resistance. In the resolution phase, production of PGE_2_ stimulates synthesis of specialized pro-resolving lipid mediators (SPMs)—lipoxins (LX), resolvins (Rv) of the E and D series, protectins (PD) and maresins (MaR)^11–13^. SPMs stop further neutrophil recruitment and stimulate infiltration of monocytes which differentiate into resolution macrophages that phagocytize and clear apoptotic neutrophils and debris via lymphatics, a process called efferocytosis, which is a key step in resolution and prevention of chronic inflammation^11,13,14^.

To date, bariatric surgery is the most effective modality to achieve remission of diabetes, but the mechanisms beyond weight loss are unclear. Our aims were to explore potential mechanisms by examining how SPM and proinflammatory eicosanoid levels change with surgical weight loss.

## Methods

### Participants

Subjects with morbid obesity who had lost > 40% of body weight at 1 year after Roux-en-Y gastric bypass (RYGB) were identified from the Geisinger prospective Bariatric Surgery biobank^15^. A control group comprised mildly obese subjects without diabetes who had stable clinical CAD. The protocol was approved by the Institutional Review Boards of the Geisinger Clinic and Beth Israel Deaconess Medical Center.

### Levels of lipid mediators: sample extraction

Serum samples stored at − 80 °C were used. Samples were extracted using protein precipitation with methanol followed by solid phase extraction (SPE) using C18 columns as described previously^16–19^. Before extraction, 500 pg of deuterium (d)-labeled internal standards, d5-MaR1, d4-RvE1, d8-5S-hydroxy-eicosatetraenoic acid (d8-5S-HETE), d4-LTB_4_, d5-lipoxin A_4_ (d5-LXA_4_), d5-maresin 2 (d5-MaR2), d4-PGE_2_ and d5-resolvin D2 (d5-RvD2), were added to facilitate quantification of analyte recovery. Then ten volumes of methanol were added, and the samples stored in the freezer at − 80° C for 1 h. After that, the precipitates were centrifuged for 10 min at 2500*g*. Sample supernatants were concentrated to 1 ml in an evaporation station (TurboVap LV, Biotage) as previously described^19^. The evaporated samples were diluted with ten volumes of ddH2O, acidified (pH ∼ 3.5) and loaded onto the C18 SPE column. Before elution, the C18 resin was washed with 6 ml of neutral ddH2O and 6 ml hexane. Samples were eluted with 6 ml methyl formate and taken to dryness using a nitrogen stream as previously described^16–19^. The extracts were suspended in methanol/water for liquid chromatography-tandem mass spectrometry (LC–MS/MS) analyses.

### Liquid chromatography–tandem mass spectrometry analysis

Targeted metabololipidomics using LC–MS/MS was performed to quantify levels of the pro-resolving SPMs (D-series resolvins, E-series resolvins, protectins and maresins) and the proinflammatory eicosanoids (PGD_2_, PGE_2_ and LTB_4_) as previously described^16–19^ using a QTrap 6500 mass spectrometer (Sciex) equipped with a Shimadzu Nexera XR HPLC system. An Agilent Poroshell 120 EC-C18 column (100 mm × 4.6 mm × 2.7 μm) was used with a gradient of methanol/water/acetic acid of 50:50:0.01 (volume/volume/volume) to 100:0:0.01 at 0.5-ml/min flow rate. A scheduled multiple reaction monitoring (MRM) method was used to monitor and quantify the levels of each lipid mediator as we previously described^19^. The MRM detected signature ion fragments for each molecule at chromatographic retention times established by a combination of external and internal synthetic standards as previously described (Supplementary Fig. S1 and Supplementary Table S1 online)^19^. Product ion spectra were collected to confirm the identity of each individual analyte by matching the data with the profiles of at least six published fragment ions as previously described^16–19^. Calibration curves were obtained using synthetic and authentic lipid mediator mixtures. Linear calibration curves for each analyte were obtained at 0.1, 1, 10, and 100 pg with r^2^ values between 0.98 and 0.99. The peak areas of the SPMs were absolutely quantified based on the linear calibration curves and the recovery rates of the internal standards. The coefficients of variation were LTB_4_: 6.2%, MaR1: 6.5%, RvE1: 11.2%, PGD_2_: 13.1% and PGE_2_: 10%. Reproducibility between laboratories and a methodological validation have been reported^20^.

### Statistical analysis

Statistical analyses were performed as previously described^21^. Normality tests were conducted using the Shapiro–Wilk test. Categorical variables were expressed as counts and percentages and compared with either Chi-square or Fisher’s exact tests. Continuous variables were reported as the mean and standard deviation (SD) for normally distributed variables or median and interquartile range [IQR] for non-normally distributed variables. Continuous variables were compared using unpaired Student’s t-tests for normally distributed variables or the Mann–Whitney-U test for non-normally distributed variables. A 2-sided *p* value ≤ 0.05 was considered statistically significant. Data analyses were performed using SPSS 20.0 for Windows (IBM Corp. Armonk, NY, USA).

### Ethical approval

All procedures performed in studies involving human participants were in accordance with the ethical standards of the institutional and/or national research committee and with the 1964 Helsinki declaration and its later amendments or comparable ethical standards.

### Informed consent

Informed consent was obtained from all individual participants included in the study.

## Results

### Baseline characteristics of subjects

The Geisinger Clinic subjects consisted of 29 subjects with morbid obesity (mean body mass index [BMI] ± SD: 53.0 ± 7.3 kg/m^2^) who had lost > 40% of their initial weight at 1 year post-bariatric surgery. Compared to 15 mildly obese (mean BMI: 30.5 ± 2.7 kg/m^2^) nondiabetic subjects, the morbidly obese subjects were significantly younger, more likely to be female and had significantly higher BMI, waist circumference and levels of insulin, triglyceride and LDL-C, presumably reflecting their greater level of obesity and associated metabolic derangements (Table 1). They also had significantly higher levels of white blood cell count (WBC) and platelets, indicating a higher level of inflammation. The morbidly obese also had a borderline significantly higher level of PGE_2_, a proinflammatory mediator, which is a signal to switch to the pro-resolving phase and bring in SPMs—PD1, RvD3 and RvD4—to resolve inflammation.

**Table 1:** Baseline characteristics for mildly obese nondiabetic subjects compared to morbidly obese subjects.

	Mild obesity (*n* = 15)	Morbid obesity (*n* = 29)	*p* value
**Demographic characteristics**			
Age (years)	63.1 ± 5.4	47.1 ± 11.0	< 0.001
Female sex, *n* (%)	1 (2.3)	27 (61.4)	< 0.001
Diabetes mellitus, *n* (%)	0 (0%)	13 (45%)	
**Anthropometric and blood pressure**			
Weight (kg)	89.3 ± 13.6	145.9 ± 21.4	< 0.001
Weight (lb)	196.9 ± 30.1	321.5 ± 47.1	< 0.001
Waist circumference (cm)	106.4 ± 7.7	144.2 ± 12.4	< 0.001
Waist circumference (inches)	41.9 ± 3.0	56.8 ± 4.9	< 0.001
BMI (kg/m^2^)	30.5 ± 2.7	53.0 ± 7.3	< 0.001
Systolic BP (mm Hg)	125.6 ± 16.1	128.5 ± 12.7	0.52
Diastolic BP (mm Hg)	74.2 ± 9.9	76.0 ± 8.8	0.55
**Biochemical profile**			
Glucose (mg/dl)	94.0 ± 8.9	111.7 ± 35.5	0.07
HbA1c (%)	5.8 ± 0.3	6.3 ± 1.1	0.07
Insulin (mIU/ml)	13.3 ± 8.1	26.8 ± 14.9	0.002
AST (IU/l)	22.1 ± 7.0	28.8 ± 19.1	0.20
ALT (IU/l)	25.8 ± 11.8	30.1 ± 17.1	0.38
WBC (10^9^ cells/l)	6.07 ± 1.53	8.51 ± 2.86	0.004
Platelet count (cells/µl)	167.2 ± 40.2	253.14 ± 86.65	0.001
**Lipids (mg/dl)**			
Total cholesterol	154.5 ± 30.7	185.3 ± 41.5	0.15
Triglycerides, median [IQR]	125.0 [71.0,188.0]	162.0 [148.0,196.5]	0.028
HDL-C	46.3 ± 12.3	43.4 ± 11.4	0.44
LDL-C	80.1 ± 23.2	105.1 ± 32.2	< 0.001
TC/HDL ratio	3.6 ± 1.4	4.4 ± 1.1	0.028

*ALT* Alanine transaminase, *AST* aspartate transaminase, *BMI* body mass index, *HbA1C* hemoglobin A1c, *HDL-C* high density lipoprotein, *LDL-C* low density lipoprotein-calculated, *TC* total cholesterol, *WBC* white blood cells.

Continuous variables are presented as mean ± SD or median [interquartile range] and categorical variables are presented as *n* (%).

A 2-sided *p* value ≤ 0.05 was considered statistically significant.

### Baseline SPM and proinflammatory eicosanoid levels before surgery

**Figure 1:**
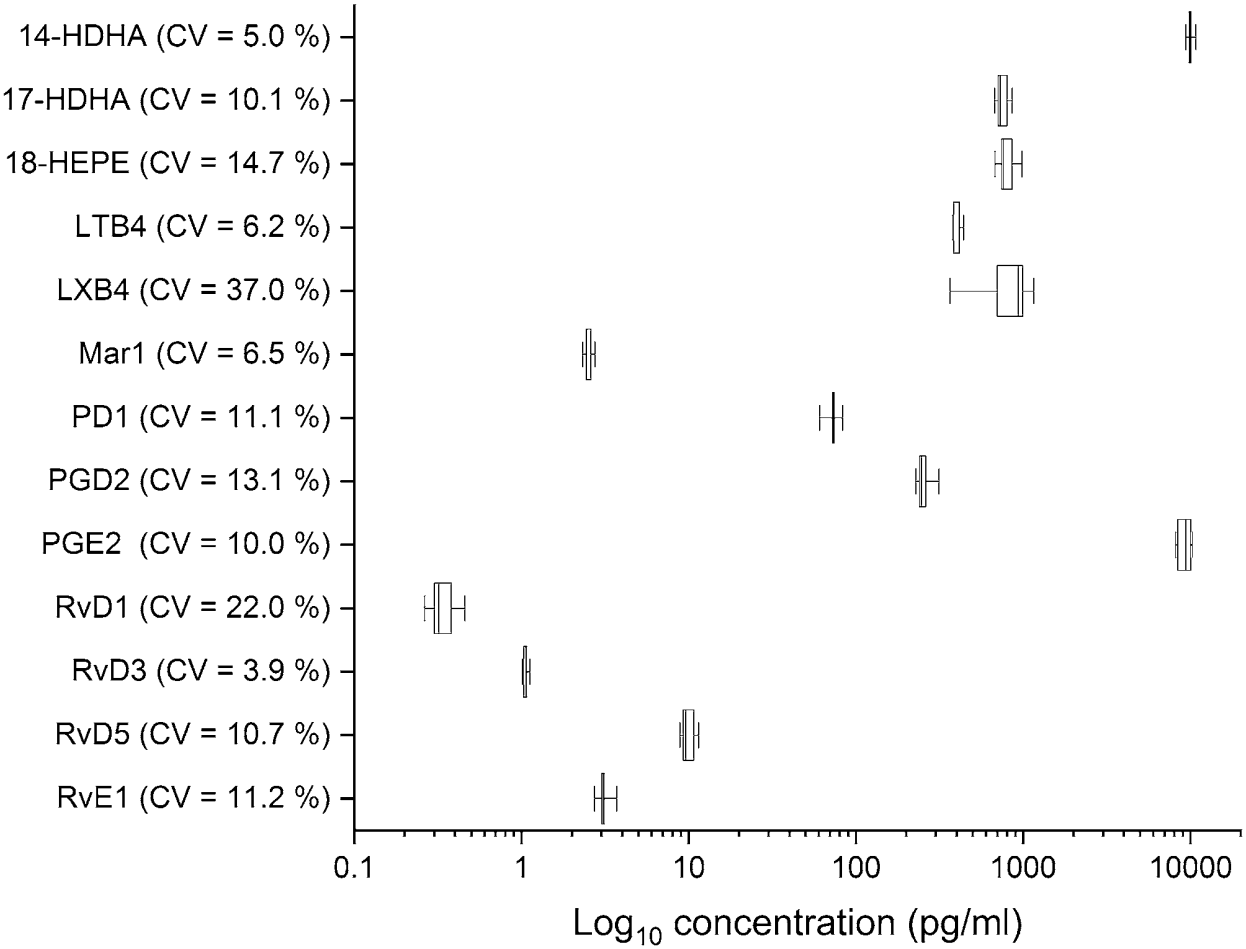
Intraday extraction and detection reproducibility for SPMs isolated from independent blood samples (n = 5). Data expressed as median [interquartile range] and whiskers showing the minimum and maximum values. *LTB*_*4*_ leukotriene B_4_, *LXB*_*4*_ lipoxin B_4_, *MaR1* maresin 1, *PD1* protectin D1, *PGD*_*2*_ prostaglandin D_2_, *PGE*_*2*_ prostaglandin E_2_, *RvD1* resolvin D1, *RvD3* resolvin D3, *RvD5* resolvin D5, *RvE1* resolvin E1, *SPM* specialized pro-resolving lipid mediators, *14-HDHA* 14-hydroxy-docosahexaenoic acid, *17-HDHA* 17-hydroxy-docosahexaenoic acid, *18-HEPE* 18-hydroxy-eicosapentaenoic acid.

Table 2 reports the lowest calibration standard on the calibration curve (LOQ) and the limit of detection (LOD) for our mass spectrometry measurements. Figure 1 reports the intraday extraction and detection reproducibility for SPMs isolated from independent blood samples (n = 5). Before surgery, compared to the 15 mildly obese nondiabetic subjects, the morbidly obese subjects had significantly higher levels of 14-HDHA (a marker of the maresin [MaR1] pathway) and the DHA-derived SPMs, PD1, resolvin D3 (RvD3) and RvD4, and PGE_2_ and significantly lower levels of 17-HDHA, a marker of the pathway for both RvD3 and PD1 and precursor for RvD3 (Table 3).

**Table 2:** Intraday extraction and detection reproducibility for SPMs isolated from independent blood samples (n = 5).

Compound	LOD^a^ (pg per injection)	LOQ^b^ (pg per injection)
d4-LTB_4_	0.5	1.0
d4-PGE_2_	0.5	1.0
d5-LXA_4_	0.1	0.2
d5-MaR2	1.0	2.0
d5-RvD2	0.1	0.2
d8-5-HETE	0.1	0.1
14-HDHA	0.1	0.1
17-HDHA	0.1	0.2
18-HEPE	0.5	1.0
LTB_4_	0.5	1.0
LXA_4_	0.1	0.2
LXB_4_	0.1	0.2
MaR1	0.2	0.5
MaR2	0.5	1.0
PD1	0.1	0.5
PDX	0.1	0.2
PGD_2_	0.1	0.1
PGE_2_	0.1	0.1
RvD1	0.2	0.5
RvD2	0.2	0.5
RvD3	0.1	0.2
RvD4	0.1	0.1
RvD5	0.1	0.1
RvE1	0.2	0.5

*d* deuterium*, LTB*_*4*_ leukotriene B_4_, *LXA*_*4*_ lipoxin A_4_, *LXB*_*4*_ lipoxin B_4_, *MaR1* maresin 1, *MaR2* maresin 2, *PD1* protectin D1, *PDX* protectin X, *PGD*_*2*_ prostaglandin D_2_, *PGE*_*2*_ prostaglandin E_2_, *RvD1* resolvin D1, *RvD2* resolvin D2, *RvD3* resolvin D3, *RvD4* resolvin D4, *RvD5* resolvin D5, *RvE1* resolvin E1, *SPM* specialized pro-resolving lipid mediators, *14-HDHA* 14-hydroxy-docosahexaenoic acid, *17-HDHA* 17-hydroxy-docosahexaenoic acid, *18-HEPE* 18-hydroxy-eicosapentaenoic acid.

^a^Limit of detection.

^b^Limit of quantitation.

**Table 3:** Lipid mediators in mildly obese nondiabetic subjects compared to morbidly obese subjects adjusted for age.

SPM (ρg/ml)	Mild obesity (*n* = 15)	Morbid obesity (*n* = 29)	*p* value
14-HDHA	36.9 [22.4, 289.6]	483.4 [170.7, 749.1]	0.011
17-HDHA	152.0 [118.1, 298.5]	90.8 [72.1, 139.0]	< 0.001
18-HEPE	67.5 [42.1, 79.0]	40.7 [27.5, 66.7]	0.040
LTB_4_	32.5 [19.2, 76.9]	19.6 [0, 72.3]	0.34
LXA_4_	0 [0, 0]	0 [0, 0.14]	0.13
LXB_4_	0 [0, 0]	0 [0, 2.0]	0.34
MaR1	24.1 [0, 41.3]	9.1 [0, 25.3]	0.69
MaR 2	0 [0, 0]	0 [0, 0]	0.14
PD1	0 [0, 0]	67.6 [55.9, 83.7]	< 0.001
PDX	0 [0, 0]	0 [0, 0]	0.40
PGD_2_	4.9 [3.6, 14.8]	4.7 [2.9, 9.1]	0.21
PGE_2_	1.7 [0, 27.6]	14.5 [2.6, 49.7]	0.054
RvD1	8.1 [0, 17.4]	5.1 [3.5, 8.8]	0.15
RvD2	0 [0, 0]	0 [0, 0]	0.63
RvD3	0.6 [0, 2.2]	2.4 [1.7, 2.9]	0.036
RvD4	0 [0, 0]	241.3 [144.3, 389.6]	< 0.001
RvD5	0 [0, 0]	0 [0, 0]	0.16
RvE1	0 [0, 0]	0 [0, 0]	0.38

*LTB*_*4*_ leukotriene B_4_, *LXA*_*4*_ lipoxin A_4_, *LXB*_*4*_ lipoxin B_4_, *MaR1* maresin 1, *MaR2* maresin 2, *PD1* protectin D1, *PDX* protectin X, *PGD*_*2*_ prostaglandin D_2_, *PGE*_*2*_ prostaglandin E_2_, *RvD1* resolvin D1, *RvD2* resolvin D2, *RvD3* resolvin D3, *RvD4* resolvin D4, *RvD5* resolvin D5, *RvE1* resolvin E1, *SPM* specialized pro-resolving lipid mediators, *14-HDHA* 14-hydroxy-docosahexaenoic acid, *17-HDHA* 17-hydroxy-docosahexaenoic acid, *18-HEPE* 18-hydroxy-eicosapentaenoic acid.

### Baseline characteristics in morbidly obese subjects stratified by diabetes status

Of the 29 morbidly obese subjects, 13 had type 2 diabetes. Before surgery, compared to the nondiabetic subjects, the diabetic subjects had significantly higher levels of glucose, 95 ± 11 mg/dl vs 131 ± 45 mg/dl, respectively, *p* = 0.02, and hemoglobin (Hb) A1c, 5.6% ± 0.4 vs 7.1% ± 1.2, respectively, *p* = 0.001 (Table 4).

**Table 4:** Baseline characteristics for morbidly obese nondiabetic subjects compared to morbidly obese diabetic subjects.

	No diabetes (*n* = 16)	Diabetes (*n* = 13)	*p* value
**Demographic characteristics**			
Age (years)	45.50 ± 8.60	49.08 ± 13.49	0.39
Female sex *n* (%)	14 (87.5)	13 (100)	0.19
**Anthropometric and blood pressure**			
Weight (kg)	143.0 ± 20.0	134.4 ± 22.9	0.67
Weight (lb)	314.5 ± 46.0	295.7 ± 43.3	0.97
Waist circumference (cm)	146.2 ± 13.5	141.6 ± 10.8	0.32
Waist circumference (inches)	57.6 ± 5.3	55.8 ± 4.3	0.32
BMI (kg/m^2^)	53.3 ± 7.6	52.7 ± 7.2	0.84
Systolic BP (mm Hg)	128.3 ± 10.1	128.8 ± 15.7	0.92
Diastolic BP (mm Hg)	76.3 ± 9.1	75.5 ± 8.8	0.82
**Biochemical profile**			
Glucose (mg/dl)	95.9 ± 10.8	131.2 ± 45.3	0.02
HbA1c (%)	5.6 ± 0.4	7.1 ± 1.2	0.001
Insulin (mIU/ml)	29.8 ± 16.2	22.7 ± 12.4	0.22
AST (IU/l)	25.1 ± 9.5	33.3 ± 26.4	0.26
ALT (IU/l)	28.9 ± 12.6	31.7 ± 21.9	0.67
**Lipids (mg/dl)**			
Total Cholesterol	190.0 ± 45.1	179.6 ± 37.5	0.51
Triglycerides, median [IQR]	162.50 [152.0, 190.8]	162.00 [127.5, 208.5]	0.92
HDL-C	44.1 ± 10.0	42.7 ± 13.4	0.75
LDL-C	110.3 ± 36.2	98.8 ± 26.5	0.35
TC/HDL ratio	4.4 ± 0.8	4.5 ± 1.4	0.68

*ALT* Alanine transaminase, *AST* aspartate transaminase, *BMI* body mass index, *HbA1C* hemoglobin A1c, *HDL-C* high density lipoprotein, *LDL-C* low density lipoprotein, *TC* total cholesterol.

Continuous variables are presented as mean ± SD or median [interquartile range] and categorical variables are presented as *n* (%).

A 2-sided *p* value ≤ 0.05 was considered statistically significant.

### SPM and proinflammatory eicosanoid levels in obese diabetic and nondiabetic subjects before and after surgery

Table 5 reports no significant differences in weight, BMI, WBC or platelet count before or after surgery in the morbidly obese nondiabetic subjects compared to the diabetic subjects. Both nondiabetic and diabetic subjects had similar significant reductions in weight (> 44% of total body weight) and BMI after surgery. WBC decreased significantly in both nondiabetic and diabetic subjects after bariatric surgery; however, platelet count decreased significantly only in nondiabetic subjects. Both WBC and platelet count are markers of inflammation and the lack of a significant reduction in platelet count in the diabetic subjects may indicate a higher level of residual inflammation post-surgery. No significant differences were observed in the proinflammatory eicosanoids and pro-resolving SPMs between the morbidly obese nondiabetic subjects and diabetic subjects before surgery, presumably reflecting higher inflammation due to excess weight in both groups. After surgery, RvD3 and PD1 and their pathway marker, 17-HDHA, were significantly lower in nondiabetic subjects compared to values before surgery, consistent with decreased inflammation. In contrast, these SPMs did not change in the diabetic subjects after surgery, presumably indicating failure to resolve inflammation despite similar weight loss. Of note, RvD1 was significantly higher and MaR1 was significantly lower in the diabetic subjects compared to nondiabetic subjects after surgery (*p* = 0.045 and 0.012, respectively).

**Table 5:** Characteristics and SPM and eicosanoid levels in morbidly obese nondiabetic subjects compared to diabetic subjects before and after bariatric surgery.

	Nondiabetic subjects (*n *= 16)			Diabetic subjects (*n *= 13)				
	Pre-surgery	Post-surgery	*p*^a^	Pre-surgery	Post-surgery	*p*^b^	*p*^c^	*p*^d^
Wt (lb)	314.5 ± 46.0	178.0 ± 27.3	< 0.001	295.7 ± 43.3	170.6 ± 26.2	< 0.001	0.97	0.76
BMI^e^	53.3 ± 7.6	28.8 ± 4.6	< 0.001	52.7 ± 7.2	28.9 ± 4.3	< 0.001	0.77	0.58
WBC^f^	8.41 ± 2.29	5.74 ± 2.08	< 0.001	8.64 ± 3.54	6.39 ± 1.75	0.007	0.46	0.58
Platelets^g^	252.1 ± 81.2	195.1 ± 75.0	< 0.001	254.5 ± 96.3	232.2 ± 125.9	0.10	0.82	0.49
**Docosahexaenoic acid metabolome (ρg/ml)**								
RvD1	4.8 [3.9, 8.5]	4.3 [3.4, 5.7]	0.33	5.4 [3.1, 8.9]	6.1 [5, 2, 12.3]	0.22	0.78	0.045
RvD3	2.5 [1.8, 3.2]	1.5 [1.3, 2.3]	0.026	2.1 [1.6, 2.7]	1.6 [1.5, 2.4]	0.60	0.42	0.45
RvD4	245.7 [145.5, 432.8]	179.9 [123.6, 426.1]	0.38	228.0 [141.1, 366.7]	188.8 [125.2, 370.8]	0.55	0.62	0.98
17 HDHA	99.9 [77.9, 153.5]	69.1 [39.5, 133.8]	0.026	88.1 [57.8, 124.0]	56.2 [42.6, 116.7]	0.60	0.37	0.95
PD1	68.9 [58.0, 83.9]	43.2 [39.6, 70.3]	0.002	64.2 [47.9, 83.7]	59.1 [39.9, 75.9]	0.65	0.56	0.50
14-HDHA	558.7 [213.5, 806.2]	345.7 [203.4, 845.2]	0.50	254.0 [56.2, 708.1]	401.1 [126.3, 942.9]	0.65	0.22	0.85
Mar1	9.5 [0, 34.2]	30.0 [1.3, 47.7]	0.43	8.8 [0, 21.2]	0 [0, 6.2]	0.21	0.81	0.012
**Eicosapentaenoic acid metabolomes (ρg/ml)**								
RvE1	0 [0, 0]	0 [0, 0]	0.32	0 [0, 0]	0 [0, 0]	1.00	0.78	1.00
18-HEPE	44.0 [26.6, 67.9]	31.2 [20.9, 53.4]	0.070	36.2 [24.4, 61.2]	34.6 [20.0, 61.2]	0.38	0.56	0.98
**Arachidonic acid metabolomes (ρg/ml)**								
LTB_4_	29.0 [0, 109.5]	22.4 [2.0, 57.2]	0.18	15.2 [0, 38.8]	0 [0, 43.0]	1.00	0.48	0.25
PGD_2_	6.3 [3.1, 8.2]	6.0 [3.2, 8.8]	0.20	4.1 [2.5, 9.6]	4.6 [2.7, 8.2]	0.70	0.62	0.56
PGE_2_	19.0 [5.1, 70.9]	37.0 [14.0, 84.0]	0.72	13.8 [0, 39.5]	16.9 [9.4, 29.6]	0.81	0.29	0.11

*BMI* Body mass index*, LTB*_*4*_ leukotriene B_4_, *MaR1* maresin 1, *PD1* protectin D1, *ρg* picogram*, PGD*_*2*_ prostaglandin D_2_, *PGE*_*2*_ prostaglandin E_2_, *RvD1* resolvin D1, *RvD3* resolvin D3, *RvD4* resolvin D4, *RvE1* resolvin E1, *Wt* weight, *14-HDHA* 14-hydroxy-docosahexaenoic acid, *17-HDHA* 17-hydroxy-docosahexaenoic acid, *18-HEPE* 18-hydroxy-eicosapentaenoic acid.

Continuous variables are presented as mean ± SD or median [interquartile range].

A 2-sided *p* value ≤ 0.05 was considered statistically significant.

^a^*p* value comparing nondiabetic subjects pre-surgery to post-surgery.

^b^*p* value comparing diabetic subjects pre-surgery to post-surgery.

^c^*p* value comparing nondiabetic subjects to diabetic subjects pre-surgery.

^d^*p* value comparing nondiabetic subjects to diabetic subjects post-surgery.

^e^BMI expressed as kg/m^2^.

^f^WBC expressed as 10^9^ cells/l.

^g^Platelets expressed as cells/µl.

### SPM and proinflammatory eicosanoid levels before and after bariatric surgery stratified by diabetes remission

Diabetes remission was defined as being off diabetic medications and an HbA1c < 6.5%^22,23^. Eight diabetic subjects had remission whereas five did not. Table 6 reports that both groups had similar reductions in weight, 44.6% vs. 46.6% and BMI, 44.8% vs. 44.9%. After surgery, the remitters had significant reductions in levels of WBC and platelet count whereas the non-remitters did not, indicating a higher level of inflammation in non-remitters. Compared to those who did not remit, those who remitted had significantly higher 14-HDHA levels (*p* = 0.019) before surgery, and they continued to produce the active SPM, MaR1, from its precursor 14-HpDHA  after surgery. After surgery, non-remitters had an 875.7% increase in 14-HDHA, (from 46 to 454 ρg/ml with the % increase significant, *p* = 0.021) whereas remitters had a 58.2% decrease (*p* = 0.030). The non-remitters had a 36.9% decrease in MaR1 to a median value of 0, suggesting that 14-HpDHA is preferentially converted to 14-HDHA rather than  to MaR1 in the non-remitters. The mean ratio of 14-HDHA/MaR1 was significantly different in remitters compared to non-remitters both pre- and post-surgery (*p* = 0.019 and 0.016, respectively) (Table 6). Thus, it appears that there is a relative impairment in the ability to produce MaR1, the active SPM, in non-remitters which may impact the anti-inflammatory, pro-resolving actions of SPMs.

**Table 6:** Pre- and post-surgery characteristics and SPM and eicosanoid levels in diabetic subjects with remission of diabetes (remitters) compared to diabetic subjects without remission of diabetes (non-remitters) after bariatric surgery.

	Pre-surgery			Post-surgery			Pre vs. Post				
	Remitters (*n* = 8)	Non-remitters (*n* = 5)	*p*	Remitters (*n* = 8)	Non-remitters (*n* = 5)	*p*	*p*^a^	*p*^b^	% change in remitters	% change in nonremitters	*p*^c^
Weight (lb)	290.0 ± 43.8	304.9 ± 45.6	0.57	167.6 ± 26.2	175.2 ± 28.4	0.63	< 0.001	< 0.001	− 44.6 ± 6.2	− 46.6 ± 4.2	0.55
BMI^d^	51.0 ± 7.6	55.3 ± 6.5	0.32	28.0 ± 4.2	30.5 ± 4.4	0.32	< 0.001	< 0.001	− 44.8 ± 7.2	− 44.9 ± 3.3	0.97
WBC^e^	8.62 ± 1.77	8.68 ± 5.67	0.97	6.00 ± 0.93	6.99 ± 2.63	0.35	0.004	0.35	− 30.1[− 43.4, − 15.9]	− 13.4 [− 39.3, 25.1]	0.58
Platelets^f^	234 ± 28	287 ± 156	0.36	195 ± 25	292 ± 198	0.19	0.016	0.83	− 21.6 [− 23.8, − 6.3]	− 3.2 [− 17.8, 13.2]	0.062
14 HDHA	439.1 [246.2, 1076.4]	46.5 [33.1, 324.7]	0.019	153.9 [109.6, 988.7]	454.1 [317.6, 1012.0]	0.28	0.58	0.14	− 58.2 [− 86.7, 118.5]	+ 875.7 [225.1, 3076.4]	0.030
MaR1	9.6 [0, 23.0]	8.8 [0, 32.6]	0.94	1.8 [0, 16.6]	0 [0, 2.8]	0.35	0.75	0.11	0 [− 80.8, 10.4]	− 36.9 [− 100.0, 0]	0.35
14 HDHA/MaR1	51.6 ± 93.4	1.7 ± 2.1	0.019	11.1 ± 14.9	45.5 ± 101.6	0.016	0.29	0.39			
17 HDHA	103.2 [71.4, 148.6]	81.6 [24.5, 91.5]	0.17	49.2 [40.7, 116.6]	99.3 [45.8, 116.7]	0.62	0.26	0.35	− 52.7 [− 67.9, 53.4]	+ 45.8 5 [− 27.6, 509.2]	0.19
18 HEPE	37.2 [31.4, 49.9]	33.4 [15.6, 105.7]	0.94	29.9 [19.9, 46.0]	35.1 [17.7, 81.6]	0.72	0.48	0.69	− 10.7 [− 63.0, 42.8]	− 46.5 [− 62.9, 318.8]	0.83
LTB_4_	21.5 [14.0, 40.1]	0 [0, 89.3]	0.17	12.1 [0, 43.1]	0 [0, 67.2]	0.72	0.89	1.00	+ 100.0 [− 100.0, 23.8]	+ 497.5 [− 100.0]^g^	0.89
LXB_4_	0 [0, 0]	0 [0, 2.2]	0.35	0.51 [0, 2.7]	2.3 [0, 3.6]	0.44	0.068	0.47			
PD1	61.7 [47.7, 84.3]	67.6 [48.8, 83.7]	0.94	64.0 [39.4, 81.5]	52.9 [38.0, 71.5]	0.62	0.89	0.23	+ 7.6 [− 30.0, 41.2]	− 24.8 [− 36.5, 6.2]	0.35
PGD_2_	3.2 [2.3, 8.3]	8.7 [3.1, 10.9]	0.44	3.2 [2.5, 13.4]	4.8 [3.8, 8.2]	0.52	0.16	0.50	+ 36.1 [− 12.2, 57.8]	− 37.6 [− 51.4, 109.6]	0.44
PGE_2_	18.8 [3.4, 55.4]	3.5 [0, 23.8]	0.22	17.2 [7.4, 30.1]	16.9 [10.5, 111.1]	0.72	0.31	0.14	− 36.4 [− 66.3, 14.8]	+ 378.0 [− 22.2]^g^	0.048
RvD1	5.3 [2.8, 9.3]	6.6 [4.0, 8.9]	0.83	7.8 [5.1, 12.7]	6.1 [4.6, 10.8]	1.00	0.40	0.35	+ 80.5 [− 34.8, 331.7]	+ 22.1 [− 6.8, 35.4]	0.44
RvD3	1.8 [1.5, 2.6]	2.5 [1.8, 3.0]	0.35	1.7 [1.4, 2.3]	1.6 [1.3, 3.1]	0.94	0.67	0.69	+ 5.8 [− 16.7, 17.0]	+ 5.1 [− 50.5, 16.2]	0.83
RvD4	221.0 [141.2, 330.5]	343.7 [74.1, 473.2]	0.62	187.9 [105.8, 382.5]	314.3 [150.2, 372.5]	0.72	0.67	0.50	− 11.6 [− 33.7, 73.1]	− 9.3 [− 31.1, − 1.5]	0.81

*BMI* Body mass index*, LTB*_*4*_ leukotriene B_4_, *LXA*_*4*_ lipoxin A_4_, *LXB*_*4*_ lipoxin B_4_, *MaR1* maresin 1, *PD1* protectin D1, *PGD*_*2*_ prostaglandin D_2_, *PGE*_*2*_ prostaglandin E_2_, *RvD1* resolvin D1, *RvD3* resolvin D3, *RvD4* resolvin D4, *WBC* white blood cells *14-HDHA* 14-hydroxy-docosahexaenoic acid, *17-HDHA* 17-hydroxy-docosahexaenoic acid, *18-HEPE* 18-hydroxy-eicosapentaenoic acid.

Continuous variables are presented as mean ± SD or median [interquartile range].

A 2-sided *p* value ≤ 0.05 was considered statistically significant.

^a^*p* value comparing pre- to post-surgery values in remitters using Wilcoxon signed Rank test.

^b^*p* value comparing pre- to post-surgery values in non-remitters using Wilcoxon signed Rank test.

^c^*p* value comparing % change in remitters to non-remitters.

^d^BMI expressed as kg/m^2^.

^e^WBC expressed as 10^9^ cells/l.

^f^Platelets expressed as cells/µl.

^g^75th quartile could not be calculated due to only three evaluable individuals.

## Discussion

In the current study, we examined the effect of morbid obesity and the effect of > 40% weight loss after bariatric surgery on levels of proinflammatory eicosanoids and pro-resolving SPMs in nondiabetic and diabetic subjects. We report several new findings. First, before surgery, the morbidly obese subjects had significantly higher levels of the DHA-derived, pro-resolving mediators, PD1, RvD3 and RvD4, compared to mildly obese subjects, presumably reflecting a compensatory response to higher inflammation in the morbidly obese. The morbidly obese also had a borderline significantly higher level of PGE_2_, a proinflammatory mediator, which is a signal to switch to the pro-resolving phase and bring in SPMs—PD1, RvD3 and RvD4—to resolve inflammation. In contrast, no significant difference was observed in the proinflammatory eicosanoids and pro-resolving SPMs in morbidly obese diabetic subjects compared to morbidly obese nondiabetic subjects before surgery, presumably due to excess weight in both groups.

A second major finding is that after surgery, the nondiabetic subjects had a significant decrease in the D-series resolvins, RvD3 and PD1, and their pathway marker, 17-HDHA, compared to pre-surgery, presumably indicating an improvement in the inflammatory state post-surgery in the nondiabetic subjects Thus, the SPM levels indicate the state of inflammation: high pre-surgery in morbid obesity with and without diabetes reflecting inflammation and diminishing SPM levels post weight loss in the nondiabetic subjects, a finding indicating a reduction in inflammation. In 197 subjects undergoing sleeve gastrectomy with BMI ≥ 35 kg/m^2^ pre-surgery, the classical inflammatory marker, high-sensitivity C-reactive protein (hs-CRP) decreased from 8.8 mg/l (0.6–45.4) pre-surgery to 2.6 mg/l (0.1–126.1) at 12 months after surgery (*p* < 0.001)^24^; therefore, the decrease in SPMs post-surgery in the current study also reflects a reduction in inflammation.

In contrast, RvD3, PD1 and 17-HDHA levels did not decrease in the diabetic subjects after surgery. This finding suggests that diabetic subjects may maintain higher levels of SPMs reflecting a continued elevated inflammatory stress due to diabetes, a proinflammatory state with elevated free fatty acid levels and continued insulin resistance. The decrease in WBC after bariatric surgery suggests a decrease in inflammation in both nondiabetic and diabetic groups as does the decrease in platelet count in the nondiabetic subjects. However, the lack of change in platelet count in the diabetic subjects is consistent with residual inflammation consistent with the unaltered SPM levels. Systemic inflammation as manifested by elevated levels of hs-CRP has been demonstrated in obesity and in type 2 diabetes^24–30^. WBC and platelet counts are also markers of inflammation and both were elevated in the morbidly obese compared to the mildly obese in the current study. Total WBC count has predicted CAD incidence and mortality in patients without CAD and predicted mortality in patients with CAD^31^. In a meta-analysis of 5337 subjects without CAD in 7 large studies, a high baseline WBC count was associated with a 1.4-fold increased risk of CAD (95% CI 1.3–1.5), comparable to that of hs-CRP (1.45, 95% CI 1.25–1.68)^32,33^. The higher WBC and platelet counts along with higher SPM levels in the morbidly obese compared to the mildly obese in the current report support an increased level of inflammation as a compensatory response to on-going inflammation.

A third major finding is the relationship between SPMs and diabetes remission. DHA is converted to 14-HpDHA which in turn is converted to either 14-HDHA or MaR1.   Before surgery, those with remission at 1 year had tenfold higher 14-HDHA levels than non-remitters and they maintained levels of the active SPM, MaR1, after surgery. In contrast, post-surgery, the non-remitters had a tenfold increase in 14-HDHA levels; however, the median value for MaR1 fell to 0, indicating a block in the conversion of the  precursor, 14-HpDHA, to MaR1 in the non-remitters. The median value for MaR1 was also 0 in the total group of diabetic subjects after surgery and significantly lower compared to the nondiabetic subjects. Perhaps higher pre-operative SPM levels of 14-HDHA  indicate higher pro-resolution capacity which might predict the ability to remit.

A prior study, the Longitudinal Assessment of Bariatric Surgery cohort, examined serum biomarkers of inflammation and adiposity as predictors of diabetes remission after bariatric surgery. In 2458 subjects, hs-CRP levels decreased more after RYBG compared to gastric banding^34^. However, neither hs-CRP nor ghrelin, leptin or cystatin C correlated with metabolic disease remission after surgery in either group. Therefore, baseline biomarkers did not predict disease remission. In the STAMPEDE trial, 150 diabetic subjects were randomized to intensive medical treatment alone, RYBG or sleeve gastrectomy. At 5-year follow-up after bariatric surgery, weight loss was 23.2 ± 9.6 kg after RYGB and 18.6 ± 7.5 kg after sleeve gastrectomy with a mean decrease in hs-CRP of 75% after RYGB and 69% after sleeve gastrectomy^35^. Remission of diabetes defined as not taking diabetic medications was 51% after RYGB and 25% after sleeve gastrectomy. These studies suggest that the level of inflammation falls after bariatric surgery due to weight loss, but there appears to be no relation between hs-CRP level and diabetes remission. In contrast, the current findings suggest that SPMs may be predictive of remission of diabetes and should be examined further in future studies.

To our knowledge, the current study is the first to report on serum levels of pro-resolving SPMs and proinflammatory eicosanoids in morbidly obese patients before and after surgically-induced weight loss and the impact of coexisting diabetes. The results suggest that SPMs may not only be a marker of inflammation but may also track the likelihood of diabetes remission. Support for this notion is found in animal models of obesity and diabetes which suggest that defective macrophage efferocytosis and apoptotic cell uptake due to free fatty acids and obesity can be reversed by the addition of SPMs. In obese diabetic mice, elevated free fatty acids prolonged neutrophil survival at a phase when neutrophils normally undergo apoptosis and are cleared from the inflammatory site^36^. Prolonged neutrophil survival leads to an increase in insulin resistance. Inflamed visceral and subcutaneous fat compartments from ob/ob obese mice, db/db obese/diabetic mice and high-fat diet-induced obese mice demonstrated deficits in RvD1 and PD1 and their pathway marker, 17 HDHA^37,38^. Consistent with this notion that defective SPM biosynthesis promotes adipose tissue inflammation, the administration of nanogram doses of RvD1 to db/db obese diabetic mice resulted in an improvement in glucose tolerance, a reduction in fasting blood glucose, an increase in insulin-stimulated Akt phosphorylation in adipose tissue and a reduction in the crown-like structures rich in inflammatory macrophages in adipose tissue^39^. Similarly, injection of nanogram amounts of RvE1 in the peritoneum of obese ob/ob mice demonstrated a significant insulin-sensitizing effect^38^. Moreover, injection of 17-HDHA, the precursor of the D-series resolvins, in the peritoneum of db/db obese diabetic mice showed a reduction in adipose tissue expression of inflammatory cytokines (MCP-1, TNF-α and IL-6), an increase in the expression of the anti-inflammatory adiponectin and an improvement in glucose tolerance which was associated with increased insulin sensitivity^38^. A lower ratio of SPM to LTB_4_ and SPM to PGs has been confirmed in omental white adipose tissue obtained from obese patients undergoing bariatric surgery^40^, a finding extending the deficit observed in animal models to the human. This imbalance may lead to an inadequate tissue resolution capacity and allow the inflammatory response to continue. Administration of RvD1 increased the IL-10 induced anti-inflammatory response and promoted the secretion of the anti-inflammatory cytokine, IL-10, in obese diabetic mice^39,41^, thus highlighting the pro-resolving functions of these lipid mediators.

Diet may play an important role as well. Compared to mice receiving a standard chow diet, aging mice (≥ 18 months old) receiving a diet enriched in omega-6 fatty acids (safflower oil-linoleic acid) had higher levels of the proinflammatory mediators, LTB_4_ and TxB_2_, and cardiac cytokines, both indicating low-grade chronic inflammation^42^. They also had increased nighttime carbohydrate utilization and atrio-ventricular block on dobutamine-induced stress test, findings suggesting an adverse effect of enrichment in dietary omega-6 fatty acids. When subjected to ligature of the coronary artery to cause myocardial infarction (MI), despite similar infarct size and left ventricular dysfunction post-MI, those on the safflower oil diet had decreased 5-, 12- and 15-lipoxygenases in the infarcted left ventricle, lower levels of pro-resolving mediators and increased expression of TNF-α and IL-1β compared to mice receiving the chow diet^43^. In contrast, feeding an omega-3 enriched diet to obese ob/ob mice resulted in increased levels of the pro-resolving mediators, RvD1, PD1 and their pathway marker, 17-HDHA, in visceral white adipose tissue^37^. Nanomolar concentrations of LXA_4_ decreased IL-6 and restored the glucose transporter, GLUT4, and IRS-1 in adipose explants from aging female mice, a finding indicating reduced inflammation and improved insulin sensitivity^44^. LXA_4_ has also been shown to preserve AKt signaling and glucose uptake in cultured adipocytes^44^. Transgenic restoration of the SPM precursors, eicosapentaenoic acid (EPA) and docosahexaenoic acid (DHA), reversed the loss of resolution capacity in adipose tissue from *fat-1* obese mice, a finding suggesting the activity of the pro-resolution SPM pathways depends on how much EPA and DHA are consumed in the diet^45^. These findings suggest that the failure of adipose tissue to produce SPMs to resolve inflammation can contribute to obesity-linked inflammation and insulin resistance. Altogether, these and our present findings strongly suggest a potential role for SPMs in obesity-linked inflammation and insulin resistance and that dietary EPA and DHA can influence the pro-resolution SPM pathways. Whether increasing SPMs in severe obesity in humans by dietary increase in EPA and DHA would favorably reduce inflammation and thereby have some clinical benefit particularly in diabetes will require further clinical study.

In conclusion, the major findings in the current study are: (1) higher levels of SPMs, WBC and platelet count in morbidly obese subjects compared to mildly obese subjects indicate a higher level of inflammation in morbidly obese subjects; (2) the decrease in SPMs in nondiabetic but not in diabetic subjects after surgery with > 40% weight loss in both is consistent with a greater reduction in inflammation in nondiabetic subjects; the lack of reduction in diabetic subjects suggests that diabetes promotes continued inflammation; and (3) the lack of remission of diabetes after surgically-induced weight loss is associated with a reduced ability to produce a specific SPM, namely MaR1. Future studies are needed to determine if SPMs are only a marker of inflammation or perhaps are, or can be, actively involved in resolving the inflammatory process in severe obesity.

## Supplementary information

Supplementary Information.

## Data Availability

All data generated or analyzed during this study are included in the published article (and its “Supplementary Information” file).
