# Supplementary material for: The relationship between specialized pro-resolving lipid mediators, morbid obesity and weight loss after bariatric surgery
Source: Sci Rep. 2020 Nov 18;10:20128. doi: 10.1038/s41598-020-75353-6 (PMC7674470; doi:10.1038/s41598-020-75353-6)
Supplement: Supplementary file 1 — Supplementary Information. [file 41598_2020_75353_MOESM1_ESM.pdf]

The Relationship between Specialized Pro-resolving Lipid Mediators, Morbid Obesity and Weight Loss after Bariatric Surgery

Fabian Schulte PhD[1,2,a], Abdul Aziz Asbeutah MD[3,a,b], Peter N. Benotti MD[4], G. Craig Wood MS[4], Christopher Still DO[4], Bruce R. Bistrian MD, PhD[5], Markus Hardt PhD[1,2] and Francine K. Welty MD, PhD[3*]

[1] Forsyth Institute, Cambridge, MA; [2] Department of Developmental Biology, Harvard School of Dental Medicine, Boston, MA; [3] Division of Cardiology, Beth Israel Deaconess Medical Center, Harvard Medical School, Boston, MA; [4] Geisinger Obesity Institute; [5] Department of Medicine, Beth Israel Deaconess Medical Center, Harvard Medical School, Boston, MA.

[a] These authors contributed equally.

[b] Current affiliation: Department of Medicine, University of Tennessee Health Science Center, Memphis, TN.

Supplementary Table S1. MRM-transitions for the detection of SPMs in human blood serum.

Compound	RT [min]	Precursor mass [m/z]	Product ions [m/z]	DP [V]	EP [V]	CXP [V]	CE [V]
d4-LTB4 (339.3/197.2)	14.2	339.3	197.2	-80	-10	-22	-22
d4-PGE2 (355.3/193.2)	11	355.3	193.2	-80	-10	-25	-25
d5-LXA4 (356.3/115.2)	11.7	356.3	115.2	-80	-10	-19	-19
d5-MaR2 (364.3/221.2)	14.6	364.3	221.2	-80	-10	-28	-28
d5-RvD2 (380.3/141.2)	11.1	380.3	141.2	-80	-10	-25	-25
d8-5S-HETE (327.2/116.1)	17.7	327.2	116.1	-80	-10	-17	-17
14-HDHA (343.2/205.1)	17.6	343.2	205.1	-80	-10	-17	-17
17-HDHA (343.2/245.1)	17.5	343.2	245.1	-80	-10	-17	-17
18-HEPE (317.2/259.1)	16.5	317.2	259.1	-80	-10	-16	-16
LTB4 (335.2/195.1)	14.2	335.2	195.1	-80	-10	-22	-22
LXA4 (351.2/115.1)	11.7	351.2	115.1	-80	-10	-20	-20
LXA4 (351.2/235.1)	11.7	351.2	235.1	-80	-10	-20	-20
LXB4 (351.2/221)	11.0	351.2	221.1	-80	-10	-20	-20
MaR1 (359.2/221.1)	14.0	359.2	221.1	-80	-10	-20	-20
MaR1 (359.2/250.1)	14.0	359.2	250.1	-80	-10	-20	-20
MaR2 (359.2/221.2)	14.6	359.2	221.2	-80	-10	-28	-28
PD1 (359.2/153.1)	13.9	359.2	153.1	-80	-10	-21	-21
PD1 (359.2/181.1)	13.9	359.2	181.1	-80	-10	-19	-19
PDX (359.1/153.1)	13.7	359.1	153.1	-80	-10	-25	-25
PDX (359.2/181.1)	13.7	359.2	181.1	-80	-10	-25	-25
PGD2 (351.3/233.1)	11.2	351.3	233.1	-80	-10	-16	-16
PGE2 (351.3/175.1)	11.0	351.3	175.1	-80	-10	-25	-25
PGE2 (351.3/189.1)	11.0	351.3	189.1	-80	-10	-25	-25
RvD1 (375.2/121.1)	11.7	375.2	121.1	-80	-10	-40	-40
RvD1 (375.2/215.1)	11.7	375.2	215.1	-80	-10	-26	-26
RvD2 (375.2/141.1)	11.1	375.2	141.1	-80	-10	-21	-21
RvD2 (375.2/175.1)	11.1	375.2	175.1	-80	-10	-30	-30
RvD3 (375.2/147.1)	11.3	375.2	147.1	-80	-10	-25	-25
RvD3 (375.2/181.1)	11.3	375.2	181.1	-80	-10	-22	-22
RvD4 (375.2/101.1)	12.7	375.2	101.1	-80	-10	-22	-22
RvD4 (375.2/255.1)	12.7	375.2	255.1	-80	-10	-25	-25
RvD5 (359.2/199.1)	13.8	359.2	199.1	-80	-10	-21	-21
RvD5 (359.2/261.1)	13.8	359.2	261.1	-80	-10	-20	-20
RvD6 (359.2/101.1)	14.3	359.2	101.1	-80	-10	-22	-22
RvE1 (349.2/161.1)	8.9	349.2	161.1	-80	-10	-25	-25
RvE1 (349.2/195.1)	8.9	349.2	195.1	-80	-10	-22	-22
RvE2 (333.3/199.1)	12.1	333.3	199.1	-80	-10	-24	-24
RvE2 (333.3/253.1)	12.1	333.3	253.1	-80	-10	-20	-20

CE Collision energy, *CXP* collision cell exit potential, *DP* declustering potential, *d4-LTB$_4$* deuterium-labeled leukotriene B$_4$, d4-*PGE2* deuterium-labeled prostaglandin E2, *d5-LXA$_4$* deuterium-labeled lipoxin A$_4$, d5-*MaR2* deuterium-labeled maresin 2, d5-*RvD2* deuterium-labeled resolvin D2, *EP* entrance potential, *LTB$_4$* Leukotriene B$_4$, *LXA$_4$* lipoxin A$_4$, *LXB$_4$* lipoxin B$_4$, *MaR1* maresin 1, *MaR2* maresin 2, *MRM* multiple reaction monitoring, *PD1* protectin D1, *PDX* protectin X, *PGD2* prostaglandin D2, *PGE2* prostaglandin E2, *RT* retention time, *RvD1* resolvin D1, *RvD2* resolvin D2, *RvD3* resolvin D3, *RvD4* resolvin D4, *RvD5* resolvin D5, *RvD6* resolvin D6, *RvE1* resolvin E1, *RvE2* resolvin E2, *SPM* specialized pro-resolving lipid mediators, *14-HDHA* 14-hydroxy-docosahexaenoic acid, *17-HDHA* 17-hydroxy-docosahexaenoic acid, *18-HEPE* 18-hydroxy-eicosapentaenoic acid, *[m/z]* mass-over-charge.

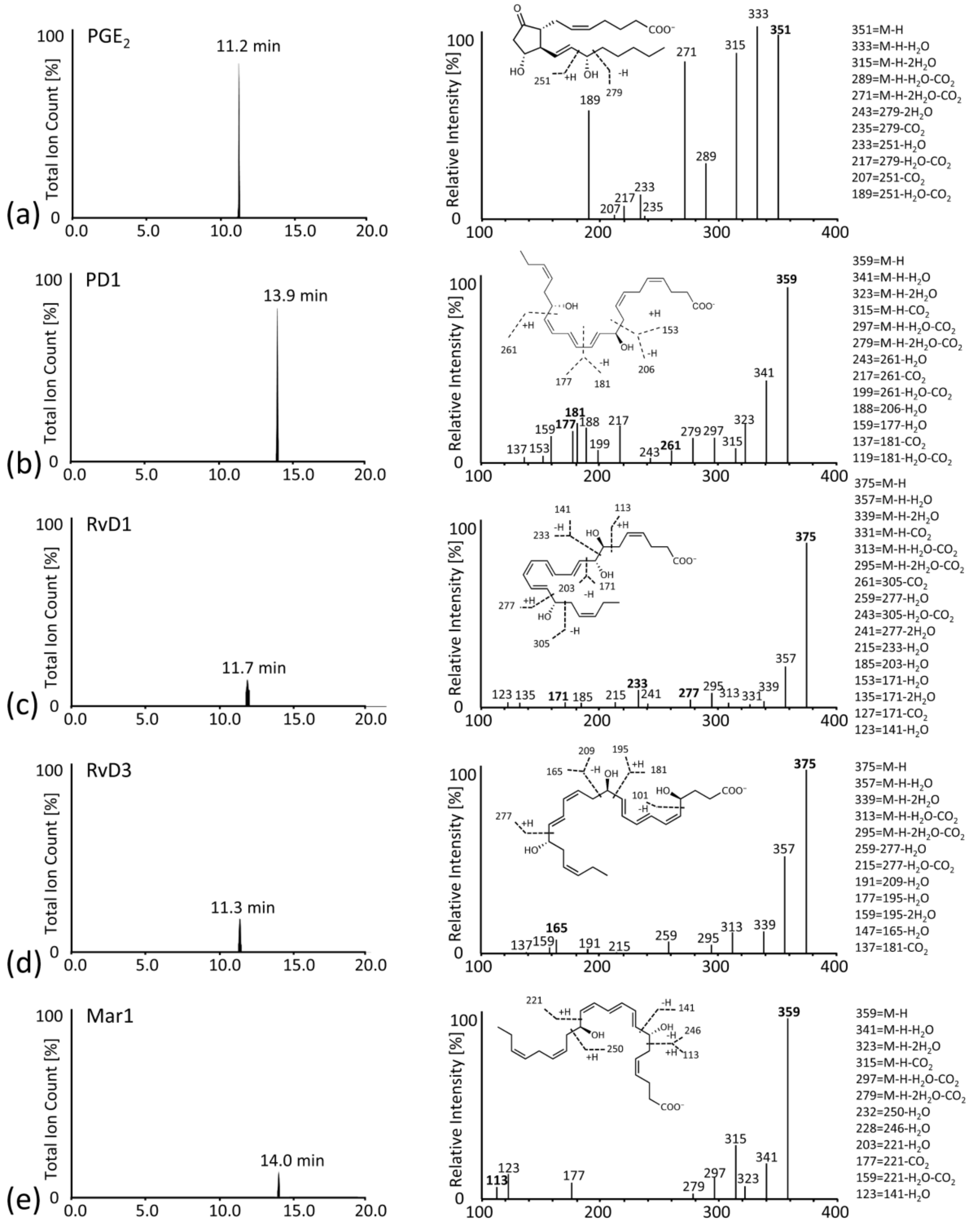

Supplementary Figure S1. Identification of lipid mediators in human blood serum.

LC-MS/MS chromatograms (left panel) and corresponding annotated product ion spectra (right panel) of the lipid mediators PGE_2 (a), PD1 (b), RvD1 (c), RvD3 (d) and MaR1 (e) isolated from a representative human serum specimen. Identification and quantification were performed by matching retention times and MS/MS-spectra of pure synthetic standards using at least 6 diagnostic ions.

LC-MS/MS Liquid chromatography tandem mass spectrometry, *MaR1* maresin 1, *PD1* protectin D1, *PGE_2* prostaglandin E2, *RvD1* resolvin D1, *RvD3* resolvin D3.
